# Supplementary material for: Immersion graded index optics: theory, design, and prototypes
Source: Microsyst Nanoeng. 2022 Jun 27;8:69. doi: 10.1038/s41378-022-00377-z (PMC9234043; doi:10.1038/s41378-022-00377-z)
Supplement: Supplementary file 1 — Supplementary material for paper ‘Immersion Graded Index Optics: Theory, Design, and Prototypes’ [file 41378_2022_377_MOESM1_ESM.pdf]

Supplementary Material for 'Immersion Graded Index Optics: Theory, Design, and Prototypes'

Nina Vaidya
Assistant Professor
Faculty of Engineering and Physical Sciences
University of Southampton
Southampton, SO16 7QF, UK
nina.vaidya@gmail.com
n.vaidya@soton.ac.uk

Olav Solgaard
Professor, Electrical Engineering
Stanford University
Stanford, CA 94305, USA
solgaard@stanford.edu

Appendix A: Scale Invariance of AGILE

Acknowledgement: scale invariance draws on Evan Scouros's: Rays in linearly graded medium

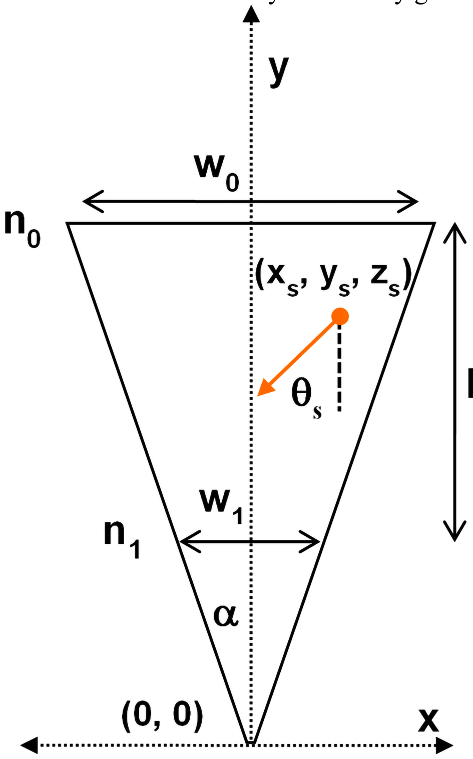

Figure A: AGILE geometry ($n_1>n_0$)

We hypothesize that the AGILE is scale invariant in the sense that if we scale the coordinates by a common factor, the traces of all rays are unaffected. To prove this hypothesis, we must show that following requirements are fulfilled: (1) reflections in the AGILE are scale invariant and that (2) the ray tracing equation for the AGILE (i.e. in a gradient index) is scale invariant.

We will use the parameters defined in Fig. A that shows a cross section of the AGILE through its optical axis. The basic AGILE is rotationally symmetric around its optical axis, but AGILEs with more complex transversal shapes can also be used. Skewed rays will behave differently in AGILEs with different transversal shapes, but all shapes are scale invariant as will become clear.

Basic Relationship:

The reflection invariance follows directly from the shape of the AGILE. The indices and widths of the input and output apertures are related as follows

$$w_1 \cdot n_1 = w_0 \cdot n_0 \qquad 1$$

In other words, the gradient index is varied such that the index*width product is constant

$$n \cdot y \cdot 2\tan\alpha = n_1 w_1 = n_0 w_0 \Rightarrow n = \frac{n_1 w_1}{y \cdot 2\tan\alpha} = \frac{n_0 w_0}{y \cdot 2\tan\alpha} \qquad 2$$

The slope of the sidewalls are given by the height and the difference in the widths

$$tan(\alpha) = \frac{\frac{w_0}{2} - \frac{w_1}{2}}{h} = \frac{w_0 - w_1}{2h} = \frac{\frac{n_1}{n_0}w_1 - w_1}{2h} \Rightarrow$$

$$\therefore tan(\alpha) = \frac{n_1 - n_0}{2h \cdot n_0} w_1 \qquad 3$$

Equation 3 proves the first of the requirements. The slope of the sidewalls, and therefore the reflection angles, are unchanged under a linear scaling in all three dimensions.
It is convenient to rewrite the index in the following form

$$n = \frac{n_1 w_1}{y \cdot 2\, tan\, \alpha} = \frac{n_1 w_1}{y \cdot 2\left[\frac{\frac{n_1}{n_0}w_1 - w_1}{2h}\right]} = \frac{n_1 n_0 h}{y \cdot (n_1 - n_0)} \qquad 4$$

Ray equation in gradient index:
We require that Snell's law applies everywhere:

$$n\, sin\, \theta = n_0\, sin\, \theta_0 = \text{Constant} = P \qquad 5$$

where θ is the angle that the ray makes with the optical axis. The starting point of the ray is at x_s, y_s, z_s, and the starting angle of the ray is designated θ_s. Our treatment is valid for all points of origin of the ray.

Snell's law tells us that the path of the ray is given by

$$tan\, \theta = \frac{d\sqrt{x^2 + z^2}}{dy} = \frac{1}{\sqrt{x^2 + z^2}} \frac{xdx + zdz}{dy} = \frac{1}{\sqrt{x^2 + z^2}} \frac{xdx}{dy} \frac{d\theta}{d\theta} \frac{dn}{dn} + \frac{1}{\sqrt{x^2 + z^2}} \frac{zdz}{dy} \frac{d\theta}{d\theta} \frac{dn}{dn}$$

$$\therefore tan\, \theta = \frac{1}{\sqrt{x^2 + z^2}} \frac{xdx}{d\theta} \frac{d\theta}{dn} \frac{dn}{dy} + \frac{1}{\sqrt{x^2 + z^2}} \frac{zdz}{d\theta} \frac{d\theta}{dn} \frac{dn}{dy} \qquad 6$$

From Snell's law

$$\frac{dn}{d\theta} = \frac{d}{d\theta}\left[\frac{P}{sin\, \theta}\right] = -P \frac{cos\, \theta}{sin^2\, \theta} \qquad 7$$

From the equation for the gradient index (Eq. 4)

$$n = \frac{n_1 n_0 h}{y \cdot (n_1 - n_0)} \Rightarrow \frac{dn}{dy} = -\frac{n_1 n_0 h}{y^2 \cdot (n_1 - n_0)} \qquad 8$$

Now we arrive at the ray equation for the AGILE

$$\frac{\frac{xdx}{d\theta} + \frac{zdz}{d\theta}}{\sqrt{x^2 + z^2}} \left(-\frac{sin^2\, \theta}{P \cdot cos\, \theta}\right)\left(-\frac{n_1 n_0 h}{y^2 \cdot (n_1 - n_0)}\right) = tan\, \theta \Rightarrow \frac{\frac{xdx}{d\theta} + \frac{zdz}{d\theta}}{\sqrt{x^2 + z^2}} = \frac{Py^2 \cdot (n_1 - n_0)}{n_1 n_0 h \cdot sin\, \theta} \Rightarrow$$

$$\int_{x_s, z_s}^{x,z} \frac{xdx + zdz}{\sqrt{x^2 + z^2}} = \frac{Py^2 \cdot (n_1 - n_0)}{n_1 n_0 h} \int_{\theta_s}^{\theta} \frac{d\theta}{sin\, \theta} \qquad 9$$

$$\int_{x_s, z_s}^{x,z} \frac{xdx + zdz}{\sqrt{x^2 + z^2}} = \frac{Py^2 \cdot (n_1 - n_0)}{n_1 n_0 h} \left[ln \frac{sin\, \theta}{1 + cos\, \theta} - ln \frac{sin\, \theta_s}{1 + cos\, \theta_s}\right] \qquad 10$$

Using Snell's law to express the ray angle in terms of the y coordinate and the initial conditions, we find the desired ray equation:

$$sin\,\theta = \frac{n_s\,sin\,\theta_s}{n} = \frac{n_s\,sin\,\theta_s}{\frac{n_1 n_0 h}{y\cdot(n_1-n_0)}} = \frac{y\cdot(n_1-n_0)\cdot n_s\,sin\,\theta_s}{n_1 n_0 h} \quad 11$$

$$\therefore \int_{x_s,z_s}^{x,z} \frac{xdx+zdz}{\sqrt{x^2+z^2}} = \frac{Py^2\cdot(n_1-n_0)}{n_1 n_0 h}\left[ln\frac{\frac{y\cdot(n_1-n_0)\cdot n_s\,sin\,\theta_s}{n_1 n_0 h}}{1+\sqrt{1-\left(\frac{y\cdot(n_1-n_0)\cdot n_s\,sin\,\theta_s}{n_1 n_0 h}\right)^2}} - ln\frac{sin\,\theta_s}{1+cos\,\theta_s}\right]$$

$$\int_{\frac{x_s}{h},\frac{z_s}{h}}^{\frac{x}{h}\frac{z}{h}} \frac{\frac{x}{n_0 w_0}\cdot d\left(\frac{x}{h}\right)+\frac{z}{h}\cdot d\left(\frac{z}{h}\right)}{\sqrt{\left(\frac{x}{h}\right)^2+\left(\frac{z}{h}\right)^2}} = P\left(\frac{y}{h}\right)^2\frac{(n_1-n_0)}{n_1 n_0}\left[ln\frac{P\left(\frac{y}{h}\right)\frac{(n_1-n_0)}{n_1 n_0}}{1+\sqrt{1-\left(P\left(\frac{y}{h}\right)\frac{(n_1-n_0)}{n_1 n_0}\right)^2}} - ln\frac{sin\,\theta_s}{1+cos\,\theta_s}\right] \quad 12$$

Equation 12 shows that the second requirement for scale invariance is fulfilled. As the AGILE is linearly scaled in all dimensions, all rays with the same initial conditions (x_s, y_s, z_s, P, $sin\Theta_s$) follow the same path in the similarly scaled x,y,z coordinates. Furthermore, we see that the scaled ray path is only dependent on the scale factor given by the height (*h*), the initial position, and the angle of the ray.

Appendix B

Ohara Glass	Refractive index at 589 nm	Linear expansion 10-7/K
S-BSL 7	1.517	75
S-TIL 1	1.548	79
S-TIM27	1.644	87
S-LAM55	1.762	71
S-LAM66	1.801	79
S-NPH53	1.847	74
S-LAH58	1.883	68
S-LAH79	2.003	60

Table B: Glasses with different refractive indices selected such that they have broadband transparency in the solar spectrum i.e., high optical transmission across the whole solar spectrum say from ~300nm to beyond ~1200nm, and similar thermal expansion and glass transition temperature so that they are compatible once bonded with each other in a stack (data and glasses supplied by Ohara Inc.).

Appendix C: Overlapping Conical Array Concentration Ratio

We calculate the exact area ratio, i.e., geometric concentration of the cluster array fabricated (input area / output area). There is difference between the nominal design as seen in Fig. C (a) and what was fabricated as seen in Fig. C (b). This difference was the result of over-cutting by the reamer as it pulled extra metal with it when machining the conical shape from the fragile metal island in between the overlapping cones at the top input surface. This was anticipated in the design and fabrication trials; and hence what was fabricated was a tile-able/tessellated structure with almost no input aperture area wasted. The CAD design of seven cones going from a radius of 3.5mm to a radius of 2mm with a spacing of 6.35mm between the centers, became seven cones of 3.6mm input radius with the same output radii and spacing.

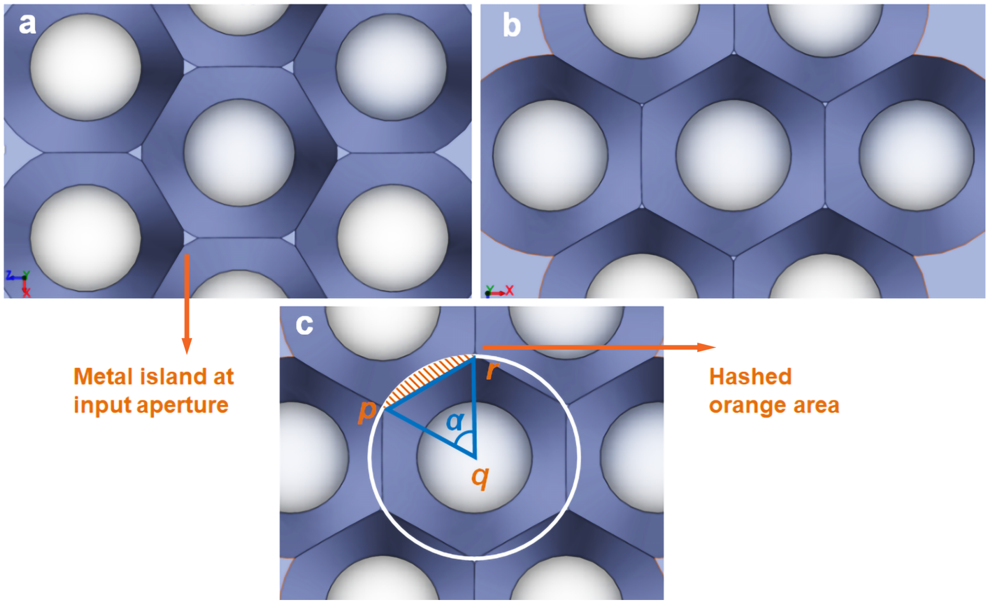

Figure C: Polymer cluster aerial view to evaluate the top surface area, (a) what was designed and (b) what was fabrication and (c) annotated figure for area calculations

We want to calculate the overlapping area, hashed orange area in Fig. C (c), in the top surface of the cones in order to calculate the total input aperture area. In this polymer cluster design, there are 7 overlapping cones which create 7 hexagon holes in the 2D plane of the top surface. To calculate the overlapping area, we will consider each arc sector and the enclosed triangle.

In the sector pqr in Fig. C (c), angle subtended by arc pqr = $\alpha = 0.9816\ rad$

Area of sector $pqr = \frac{r^2\Theta}{2} = 6.3611mm^2$

Area of triangle $pqr = \frac{bh}{2} = 5.3875mm^2$

Overlapping area between two neighbouring sectors = 2 x hashed orange region = 2 x (Area of sector pqr - Area of triangle pqr)

$$= 2(6.3611 - 5.3875)mm^2$$

$$= 1.9471mm^2 \approx 1.95mm^2$$

12 such overlapping areas for a cluster of 7 circles $= 12 \times 1.95mm^2 = 23.40mm^2$

Total input aperture area = area of 7 circles – overlapping area calculated above

$= (7 \times \Pi \times 3.6^2) - (23.40) = 261.61mm^2$

Concentration ratio = input area calculated above / area of 7 smaller circles at the output $= \frac{261.61mm^2}{(7\times\Pi\times2^2)mm^2} = 2.97$

Therefore, concentration of the cluster after fabrication = 2.97

Appendix D

Creating a graded index stack using polymers:

1. Material search was done across various types of optically transparent polymers: silicones, acrylate polymers, polyimides, and polyurethanes (heat and/or UV curable resins). Ellipsometer and spectrophotometer measurements were done for various optical polymer film samples made of a fixed thickness to characterize the properties by measuring the film transmission and RIs across the solar spectrum. Polymers with broadband transparency (i.e., high optical transmission across the whole solar spectrum say from ~300nm to beyond ~ 1200nm) and having refractive indices evenly distributed in as large an index range as possible were chosen.

2. UV curable optical polymers from Norland Products with RIs 1.46, 1.51, 1.52, 1.54, 1.56, and 1.625 were chosen for fabricating the AGILEs. The single AGILE and 10 layer cluster were made using polymers with index 1.46, 1.51, 1.52, 1.54, and 1.56. The RI=1.625 layers, which require curing in an inert atmosphere (glovebox) was used in the 12-layer cluster fabricated.

3. Molds were made by reaming cone shaped cavities in aluminium metal plates. These cavities were polished using decreasing grit size sandpapers and polishing agents to make them optically reflective. After cleaning steps, a flat PDMS (Polydimethylsiloxane) film was attached using water soluble glue at the edges at the bottom of the AGILE reflective mold to seal the output in order to start filling in the graded index polymer layers (the UV curable optical polymers do not stick to PDMS and at the end of fabrication the device can be peeled off from the PDMS substrate). If curing is done first from the top there is the

issue of uncured gel trapped below a cured top crust. To ensure a complete cure from the base, curing was first done with UV light incident through the PDMS layer from below the device, which was placed on a stand. . Later the cure was finished off from the top.

4. The UV cure parameters were tuned, such as, power and wavelength of the source, distance from the source, and the duration of cure. Some layers were fabricated in a glovebox due to need for inert atmosphere during the cure to avoid yellowing/clouding in air, e.g., oxygen inhibition in some of the polymers . Air gaps and bubbles were removed pre-cure by intermediate vacuum treatment steps. Some polymers shrunk during the cure and some needed age hardening after cure to achieve the required transparency and RI. These material differences were taken into account to make uniform and broadband transparent layers. Compatibility of the polymers with their neighbouring layers in the stack sequence was also tested before final fabrication.

5. Layers were filled volumetrically using pipettes to have a fixed layer height in order to complete the conical geometry. Each layer was filled and cured in several steps to ensure thin layers and hence a complete cure. UV curable polymer layers were formed one at a time starting from low-index to high-index polymers in order to fill half of the back-to-back shape; this process was followed by filling the upper part of the AGILE with high-index to low-index polymers. The single AGILE was filled from high-index to low-index. This completed the multi-stage polymer deposition and curing steps to create a graded index material with a large index variation.

Appendix E

Characterizing AGILE using HeNe laser

1. Test set up for measuring the light concentration ability included a red laser (HeNe Laser, 632.8 nm, 0.5 mW), beam expander, and a rotational stage as seen in Fig. 6a in the manuscript. Using a 3D printed holder the AGILE was fixed flat on the solar cell/photodetector which was mounted on a rotational stage. The AGILE was secured on the photodetector with both mechanical and optical bonding. 3D printed holders were painted black so that stray light is absorbed and only light going through the AGILE reaches the detector. The beam expander was used so we over fill/illuminate the input aperture uniformly. For accuracy of the measurements, the center of the input plane of the AGILE, i.e., input aperture was fixed at the center of rotation of the stage. The measurements have full 360º symmetry (important for solar applications) for axially symmetric shapes of the AGILEs tested like the over lapping polymer cluster. For the pyramid which is not axially symmetric two measurements were taken- rotation done along the side (0°) and rotation done along the diagonal (45°) of the square input aperture of the pyramid.

2. A visible wavelength laser is an ideal source to test the optical concentration efficiency of AGILE as it provides control over the incidence angle and also uniform area coverage at the input aperture. The current-voltage readings from the photodetector circuit were measured at different incidence angles (0 to 89°) of light through the AGILE to represent the light concentrated at the output. These values were compared to the readings without the AGILE but with the same input aperture on the photodetector to create normalized transmission curves. This allowed us to evaluate the effectiveness of AGILE by comparing (a) light collected by AGILE from a fixed input aperture area onto a 3x smaller photodetector area at the output; with (b) light collected on the same fixed input aperture area on the photodetector without AGILE. The results graph is drawn from average values, each current-voltage reading was taken several times and the mean values were calculated. To make the results as directional as possible, measurements were taken with background lights off and the AGILE was placed at a fixed distance away from the source.
